# Making it to the PhD: Gender and student performance in sub-Saharan Africa

**DOI:** 10.1371/journal.pone.0241915

**Published:** 2020-12-14

**Authors:** Monica Fisher, Violet Nyabaro, Ruth Mendum, Moses Osiru

**Affiliations:** 1 Social Science and Impact Assessment Unit, International Centre of Insect Physiology and Ecology, Nairobi, Kenya; 2 Social Science and Impact Assessment Unit, International Centre of Insect Physiology and Ecology, Nairobi, Kenya; 3 Office of International Programs, College of Agricultural Sciences, The Pennsylvania State University, Philadelphia, PA, United States of America; 4 Regional Coordination Unit of the Regional Scholarship and Innovation Fund, International Centre of Insect Physiology and Ecology, Nairobi, Kenya; Georgia Southern University, UNITED STATES

## Abstract

Women’s underrepresentation in science, technology, engineering, and mathematics (STEM) impedes progress in solving Africa’s complex development problems. As in other regions, women’s participation in STEM drops progressively moving up the education and career ladder, with women currently constituting 30% of Africa’s STEM researchers. This study elucidates gender-based differences in PhD performance using new survey data from 227 alumni of STEM PhD programs in 17 African countries. We find that, compared to their male counterparts, sampled women had about one less paper accepted for publication during their doctoral studies and took about half a year longer to finish their PhD training. Negative binomial regression models provide insights on the observed differences in women’s and men’s PhD performance. Results indicate that the correlates of publication productivity and time to PhD completion are very similar for women and men, but some gender-based differences are observed. For publication output, we find that good supervision had a stronger impact for men than women; and getting married during the PhD reduced women’s publication productivity but increased that of men. Becoming a parent during the PhD training was a key reason that women took longer to complete the PhD, according to our results. Findings suggest that having a female supervisor, attending an institution with gender policies in place, and pursuing the PhD in a department where sexual harassment by faculty was perceived as uncommon were enabling factors for women’s timely completion of their doctoral studies. Two priority interventions emerge from this study: (1) family-friendly policies and facilities that are supportive of women’s roles as wives and mothers and (2) fostering broader linkages and networks for women in STEM, including ensuring mentoring and supervisory support that is tailored to their specific needs and circumstances.

## Introduction

Africa’s development challenges include how to increase agricultural productivity, foster equitable economic growth, reduce environmental degradation, achieve food and nutrition security, and tackle the triple disease burden (non-communicable, communicable, and reproductive health related). Advances in science, technology, engineering, and math (STEM) fields are essential to finding effective solutions to Africa’s complex development problems and will require harnessing the continent’s human resources, both women and men [1–3]. When a greater diversity of perspectives is engaged in scientific and technical endeavours, conventional assumptions are challenged, scientific findings are more complete and robust, and STEM innovations address the demands and circumstances of a diversity of stakeholders [2–4].

**Fig 1:**
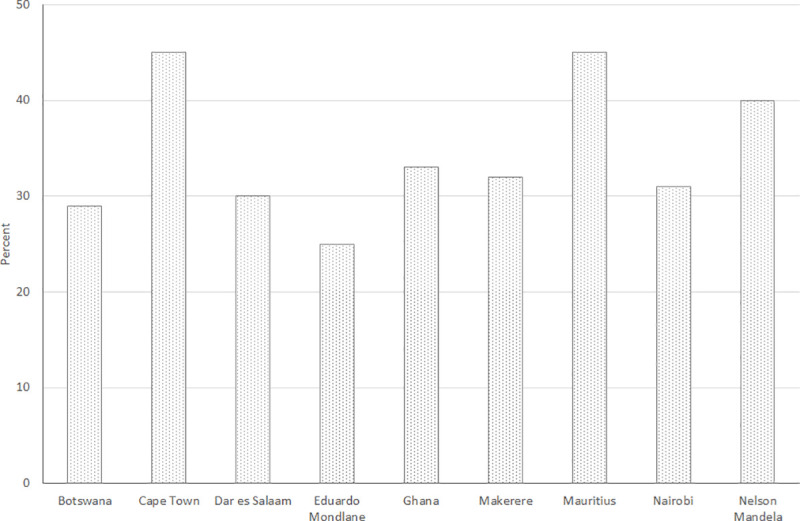
Percent women students enrolled in science and technology fields in 2010/11: Undergraduate and postgraduate levels at nine selected African universities. Source: Bunting et al. (2014) [6].

**Fig 2:**
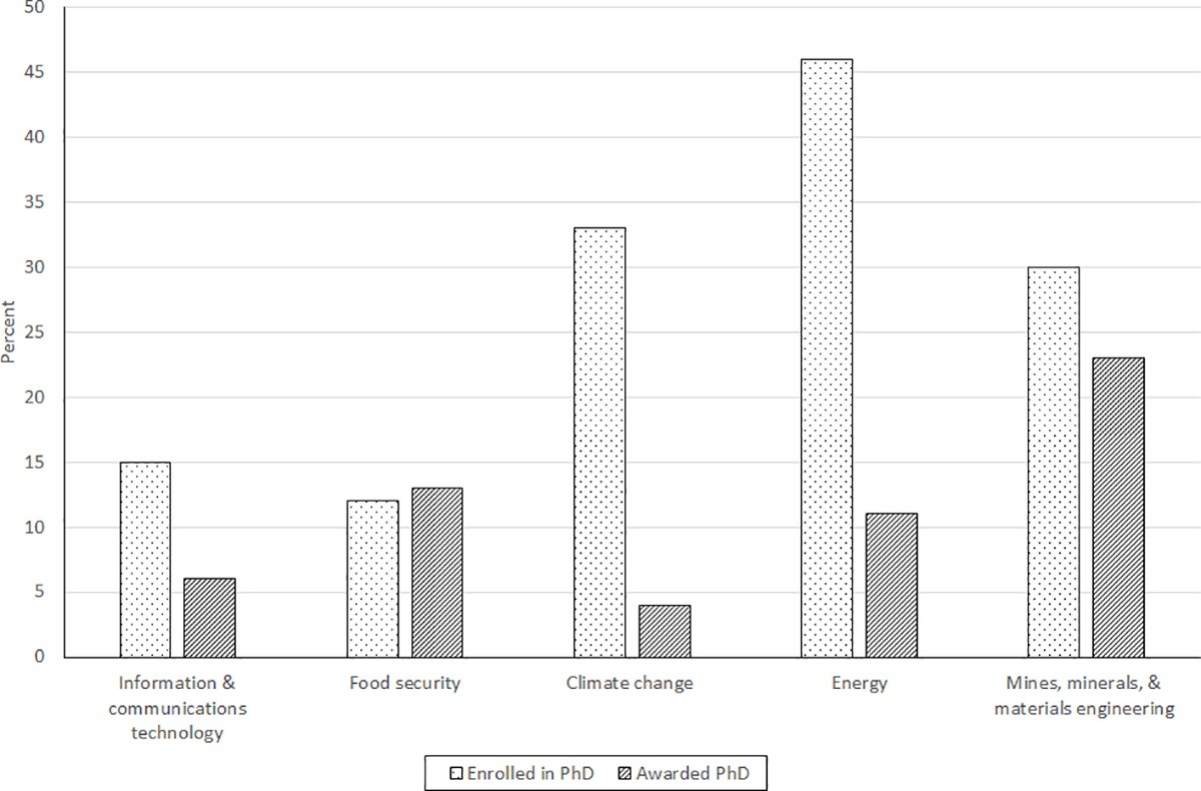
Percent of women enrolled in doctoral programs and awarded PhDs by RSIF theme, averages 2015/16–2018/19. Source: Enrollment and completion data provided by nine of the RSIF’s 11 African Host Universities (AHUs), 2019/2020. University of Felix Houphouet-Boigny, Cote D’ Ivoire; University of Ghana; Kenyatta University, Kenya; University of Nairobi, Kenya; African University of Science and Technology, Nigeria; University of Port Harcourt, Nigeria; Bayero University, Nigeria; University of Rwanda; Nelson Mandela University of Science and Technology, Tanzania; Sokoine University of Agriculture, Tanzania; and University of Gaston Berger, Senegal.

Unfortunately, available evidence indicates that only a fraction of women’s potential contributions is currently being harnessed. Women make up 30% of researchers in science fields in sub-Saharan Africa (SSA), roughly the same as the global average of 28% [5]. Recent data [6] from nine flagship African universities for 2010/11 show female student enrollment in undergraduate and postgraduate STEM fields ranges from a low of 25% (Edward Mondlane University) to a high of 45% (Cape Town University and University of Mauritius) (Fig 1). Not revealed by Fig 1 is that female representation drops progressively moving up the education (and career) ladder. In Africa, women represent over half the science graduates at Bachelor’s level (53%), compared to 43% at the Master’s level and 28% at the PhD level [5]. Fig 2, based on recent data for nine doctoral programs in seven countries in West and East Africa, shows that women’s representation in STEM varies considerably across disciplines, a common finding in the literature [7–9], although gender parity is not observed in any of the represented STEM groupings.

A complex interaction of many factors explains women’s underrepresentation in science and technology fields, with the STEM pipeline “leaking” girls and women at various stages from secondary school through undergraduate and graduate studies and as they transition to a career in STEM [4,10–16]. The present study focuses on the PhD portion of the STEM pipeline and examines whether there are gender-based differences in PhD performance (e.g., number of publications) and completion for students in SSA. The study is based on survey data collected in 2020 from 227 individuals (72% female) who pursued a STEM PhD in the last 20 years at a university in SSA. The data covers 27 disciplines, 40 universities, and 17 African countries. We apply a broad definition of STEM that includes, along with formal and natural sciences, the social sciences, specifically economics and psychology, both of which are critical to understanding applied issues such as food security.

Regression analysis uses the retrospective data to elucidate the key socio-cultural, economic, and institutional factors that influence PhD students’ performance (measured as number of peer-reviewed publications) and timely completion of the PhD (measured as the number of years taken to complete the PhD). We first use regression analysis to identify the common factors that are associated with PhD performance and completion for women and men. Next, interaction terms are included in the models to elucidate gender differences in influential factors. Specification of the empirical models relies on review of a large body of literature [7,15,17] that examines the key implicating factors in the female STEM shortage at the PhD level and beyond.

The key contribution of this study is the geographical focus on Africa. Nearly all studies in the extensive literature on women in STEM concern North America and Europe, a knowledge gap this study helps to address. Available data suggests that gender gaps in STEM vary considerably across regions [5], and chief barriers to women in STEM or best approaches to remove those barriers are likely to differ substantially between SSA and other regions as well as within SSA. Influential commentaries and reviews [2,3] stress the urgency of closing Africa’s gender gaps in STEM, and there is an emerging rigorous qualitative literature, e.g., [12,18–21]. However, we know of only two quantitative studies on women’s participation in STEM in SSA [14,22]; both are descriptive rather than inferential. A second important contribution of our study is the set of evidence-based interventions we present that universities and other institutions could adopt to enhance women’s participation in PhD programs in STEM fields in SSA.

The remainder of the study is organized as follows: The next section describes the dataset and empirical modeling approach. This is followed by the results section, which includes both descriptive statistics and regression model results. The discussion section synthesizes the study’s key findings, considers how the results agree or differ with previous research, describes study limitations, and recommends feasible policies and practices for advancing women in STEM in SSA.

## Materials and methods

### Survey sampling and data collection

The data for this study was collected as part of a mixed-methods (qualitative and quantitative) research study undertaken by the Regional Scholarship and Innovation Fund (RSIF) with the objective of developing a gender strategy for the program. RSIF is one of the flagship programs of the Partnership for Skills in Applied Sciences, Engineering and Technology (PASET), an Africa-led initiative with the objective of strengthening the applied science, engineering, and technology (ASET) capability in Africa to further its socio-economic transformation. PASET was launched in 2013 by the governments of Senegal, Ethiopia, and Rwanda with facilitation by the World Bank. Other governments including Kenya, Cote D Ivoire, Ghana, and Burkina Faso have since joined the initiative. RSIF has the following objectives: (1) Create a stock of highly skilled scientists, professionals and innovators in ASET areas; (2) Identify and nurture young talented Africans to further their studies in ASET fields where expertise is needed most; (3) Address imbalances in the number of women and disadvantaged groups in ASET fields in Africa; and (4) Build African university capacity to provide relevant ASET training and to ensure continued investment in scaling up ASET education and workforce. The program seeks to achieve gender parity among its PhD scholars.

This research study was approved by the Institutional Review Board of the International Centre of Insect Physiology and Ecology (*icipe*). The study was approved because (a) it was a socioeconomic study that collected data through an online SurveyMonkey survey with adult respondents (minors were not involved in the study, no blood samples were taken from humans, and no animals were involved), (b) the research questions were deemed as well defined and analysis methods considered sound, (c) the study protocol outlined clear strategies for protecting the privacy of the survey participants via data anonymization, and (d) the online survey instrument provided an informed consent form (described below).

The online survey was completed by 163 women and 64 men who had pursued a STEM PhD at a university in SSA in the last 20 years. Probability sampling of respondents was not possible, given the lack of a sample frame (i.e., a list of all recent PhD students in STEM at SSA universities). Survey participants were solicited by posting the survey link on the RSIF website and sharing it with African-university professors known by the study’s authors, representatives of organizations working to advance women in STEM (e.g., Mawazo Institute and Portia) who in turn shared it widely within their networks, and former PhD students who had attended one of the 11 RSIF African host universities (AHUs). The survey link specified that we sought participants meeting the following criteria: former PhD student in a STEM field at an African university that exited the PhD program (with or without a degree) in the last 20 years.

The survey had French and English versions and collected data on a range of variables reflecting demographics, socioeconomic status, PhD funding sources, motivation for pursuing a doctorate, psycho-social wellbeing during the PhD training, perceptions of gender stereotypes and discrimination in the PhD program, university resources (e.g., scientific writing course offered and presence of a gender and diversity office), PhD performance, PhD completion, and persistence in STEM. The first page of the survey was a standard informed consent form that described the voluntary nature of the survey, data confidentiality, any potential risks and benefits, the expected survey duration, and the types of information sought. Respondents had to agree to the consent form electronically in order to continue to the survey questions.

**Fig 3:**
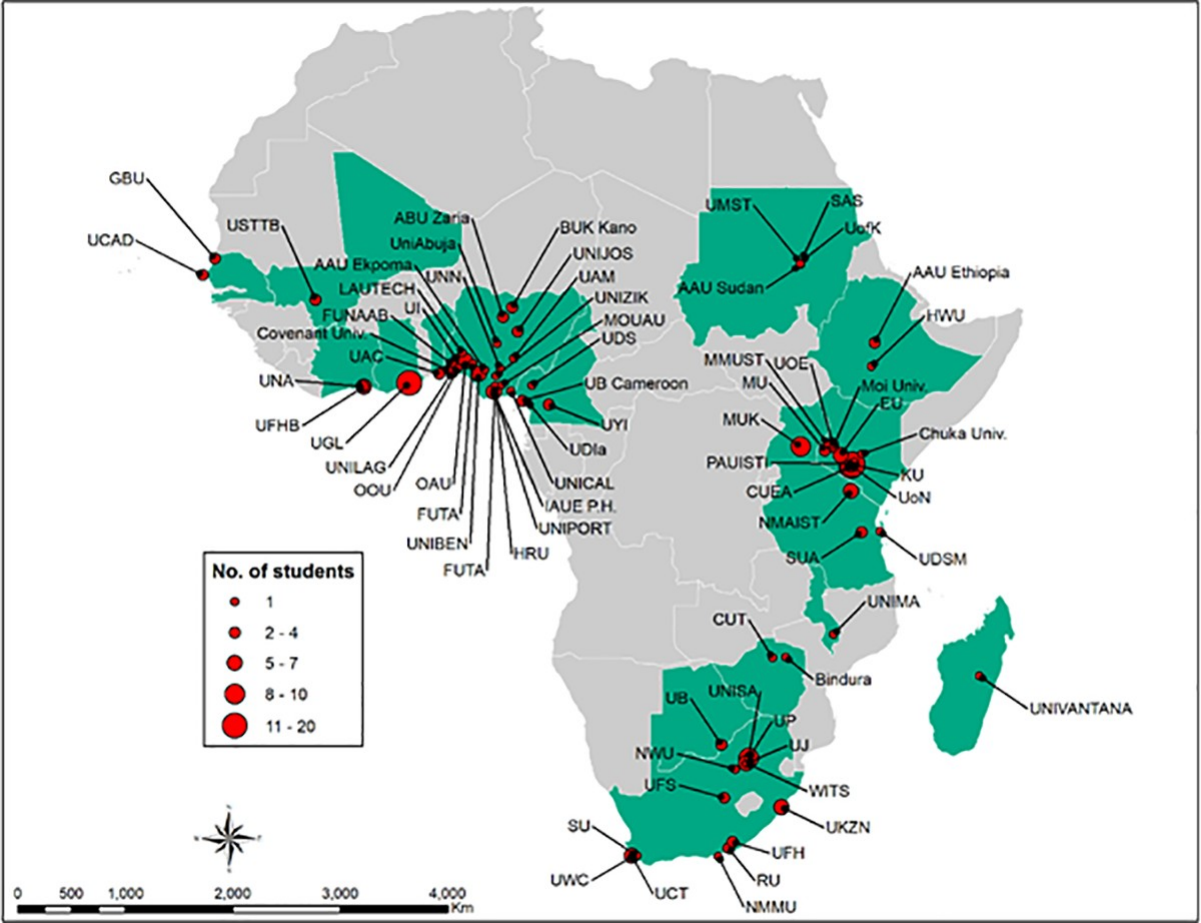
Map of the countries and institutions included in the sample. The map was developed using QGIS 3.10.9 software (https://www.qgis.org). The administrative boundaries incorporated in the map were sourced from Natural Earth (http://www.naturalearthdata.com/). The co-ordinates for the universities were obtained from Google maps (https://www.google.com/maps/).

The initial sample size was 262 individuals that completed the survey, which later reduced to 227 after removal of respondents from universities outside SSA. Fig 3 shows the spatial distribution of the sample, covering 17 countries in West Africa, East Africa, and southern Africa.

### Empirical method

We use multiple regression analysis to examine the main factors associated with two outcome variables: number of publications accepted at peer-reviewed journals during the PhD training and number of years taken to complete the PhD. The basic regression equation is of the following form:

$$Y=\beta_{o}+\beta_{1}X+\beta_{2}F+\beta_{3}S+\beta_{4}P+\beta_{5}I+\beta_{6}D+\beta_{7}R+\beta_{8}T+\varepsilon_{i}.$$

In eq 1 above, *Y* is alternately number of publications and years to PhD completion. The regressions are estimated with the negative binomial model, given the dependent variables are counts. We chose the negative binomial model over the poisson model, given the former model’s advantage in handling over-dispersion [23], which is present in our data. We had initially intended to include as an outcome variable whether the respondent completed the PhD. However, non-completion was a rare event among our sample, which presents difficulties for explanation and prediction in regression analysis [24].

The models are specified slightly differently (e.g., number of predictors and how variables are measured) for number of publications and years to PhD, but the sets of explanatory variables are the same across regression models. In the equation, vector ***X*** denotes the PhD alumni’s personal characteristics (age at the time of entering the PhD program and gender); ***F*** represents family-related factors (binary variables for entering into marriage or a marriage-like relationship and becoming a parent during the PhD training); *S* indicates that university or non-university funding was the main source of PhD financing; ***P*** are psycho-social variables (being motivated by an excellent research opportunity, having a personal goal for timely PhD completion, and having a supervisor that provided regular professional guidance and was supportive); vector ***I*** includes institutional factors (binary variables for whether the student participated in an orientation program or a writing course and if the university had a time limit for PhD completion); ***D*** is a set of binary variables for the PhD discipline: social science (economics and psychology) and a category combining formal science (mathematics and statistics), engineering, and information and communications technology (ICT) due to very small numbers of observations for engineering and ICT (reference category is natural science); ***R*** is region of the PhD institution; and *T* is the year of exit from the PhD program.

In addition to regression analyses for the pooled sample of women and men PhD alumni, we estimated models for number of publications and years to PhD completion for the women sub-sample. This allowed us to investigate the influence of factors that either did not vary for men (none of the sampled men had a female PhD supervisor) or were not expected to influence men’s PhD outcomes. The women-only regressions include three additional variables: a binary variable for female supervisor, whether the student knew of any policies and practices in support of women students at their PhD institution, and the student’s perception of the frequency of sexual harassment by faculty in their PhD program.

The choice of independent variables in the models is based on a review of empirical studies on the main factors associated with the study’s focal outcomes variables: publications during the PhD training [25–27] and time to PhD completion [28–31]. Table 1 presents means and 95% confidence intervals for model variables by student gender. The second column of the table indicates in which outcome model each explanatory variable is included, where *Y*_*1*_ is the number of publications accepted during the PhD training and *Y*_*2*_ is the number of years the student took to complete the PhD. Notes at the bottom of Table 1 provide definitions for those explanatory variables that require further explanation.

**Table 1:** Descriptive statistics for regression model explanatory variables.

Explanatory variables	Outcome models	Women (*n* = 163)			Men (*n* = 64)		
		Mean	Lower 95% CI	Upper 95% CI	Mean	Lower 95% CI	Upper 95% CI
Age (years) at start of PhD training	*Y*_*1*_, *Y*_*2*_	33.037	32.051	34.022	33.111	31.827	34.395
Got married during PhD training (0/1)	*Y*_*1*_, *Y*_*2*_	0.209	0.146	0.272	0.172	0.077	0.267
Birth/adoption of child during PhD (0/1)	*Y*_*1*_, *Y*_*2*_	0.147	0.092	0.202	0.219	0.115	0.323
University or non-university funding (0/1) ^a^	*Y*_*1*_, *Y*_*2*_	0.558	0.481	0.635	0.906	0.833	0.980
Excellent research opportunity (0/1) ^b^	*Y*_*1*_	0.399	0.323	0.475	0.219	0.115	0.323
Goal for timely PhD completion (0/1)	*Y*_*2*_	0.681	0.609	0.753	0.750	0.641	0.859
Advisor provided good guidance (0/1) ^c^	*Y*_*1*_, *Y*_*2*_	0.595	0.519	0.671	0.641	0.520	0.761
Female PhD supervisor (0/1)	*Y*_*1*_, *Y*_*2*_	0.307	0.235	0.378	0.000	0.000	0.000
Sexual harassment perceived as common (0/1) ^d^	*Y*_*1*_, *Y*_*2*_	0.086	0.042	0.129	0.047	-0.006	0.100
University time limit for PhD (0/1)	*Y*_*2*_	0.325	0.252	0.398	0.422	0.298	0.546
Participated in writing course (0/1)	*Y*_*2*_	0.644	0.570	0.718	0.656	0.537	0.776
University had gender policies (0/1) ^e^	*Y*_*1*_, *Y*_*2*_	0.245	0.179	0.312	0.219	0.115	0.323
Participated in orientation (0/1)	*Y*_*1*_	0.405	0.329	0.481	0.422	0.298	0.546
Natural science (0/1, reference category)	*Y*_*1*_, *Y*_*2*_	0.767	0.701	0.832	0.797	0.696	0.898
Formal science, engineering, and ICT (0/1)	*Y*_*1*_, *Y*_*2*_	0.080	0.038	0.122	0.188	0.089	0.286
Social science (0/1)	*Y*_*1*_, *Y*_*2*_	0.153	0.097	0.209	0.016	-0.016	0.047
East Africa (0/1) ^f^	*Y*_*1*_, *Y*_*2*_	0.405	0.329	0.481	0.344	0.224	0.463
West Africa (0/1) ^g^	*Y*_*1*_, *Y*_*2*_	0.491	0.413	0.568	0.219	0.115	0.323
Southern Africa (0/1, reference category) ^h^	*Y*_*1*_, *Y*_*2*_	0.104	0.057	0.152	0.438	0.313	0.562
Year left PhD program	*Y*_*1*_, *Y*_*2*_	2016	2016	2017	2015	2014	2016

a. University of non-university funding was the most important source of financial support during the PhD.

b. Respondent agreed or strongly agreed with the statement that she/he had the opportunity during the PhD training to work on cutting-edge research or with a prestigious faculty member.

c. Respondent reported that she/he had a PhD advisor who provided effective guidance in professional activities (e.g., writing articles for publication, conference presentation, networking) and was supportive of the student’s personal career goals, and that the student and advisor met frequently or often enough during the PhD training.

d. Respondent agreed or strongly agreed that sexual harassment by faculty was a common occurrence for women students in their PhD program.

e. Respondent’s reply to a yes/no question of whether the university of their PhD training had any policies and practices in place to support women graduate students.

f. East Africa includes Ethiopia, Kenya, Sudan, Tanzania, and Uganda.

g. West Africa includes Benin, Cameroon, Ghana, Ivory Coast, Mali, Nigeria, and Senegal.

h. Southern Africa includes Botswana, Madagascar, Malawi, South Africa, and Zimbabwe.

## Results

### Descriptive statistics

Table 1 provides a description of our sample of individuals that pursued a PhD in a STEM field at an African university between 2000 and 2020. For many of the explanatory variables, numerical differences in means between women and men PhD alumni are small and not statistically significant. For instance, sampled individuals had an average age of 33 years at the start of their PhD training, about 20% entered marriage or a marriage-like relationship during their doctoral studies, and about 40% participated in an orientation program during their PhD training. A few gender-based differences stand out. Women were far less likely than men to have university or non-university funding as their main source of PhD financing. Not shown in the table is that other main sources of PhD funding for the sampled women were self-financing and parental support; financial support by spouses was relatively unimportant. Among the interviewees, none of the men and 30% of women had a female PhD advisor. Interestingly, women in our study were more likely to report that they had an excellent research opportunity during their PhD training. The survey data also show that sampled women had greater representation in social science than men, which is consistent with global trends [10]. Women were more likely than men to have pursued their PhD in West Africa and less likely to have studied in southern Africa. The latter is likely a feature of our non-random sampling design rather than a true reflection of spatial distribution of women and men PhDs across SSA.

**Fig 4:**
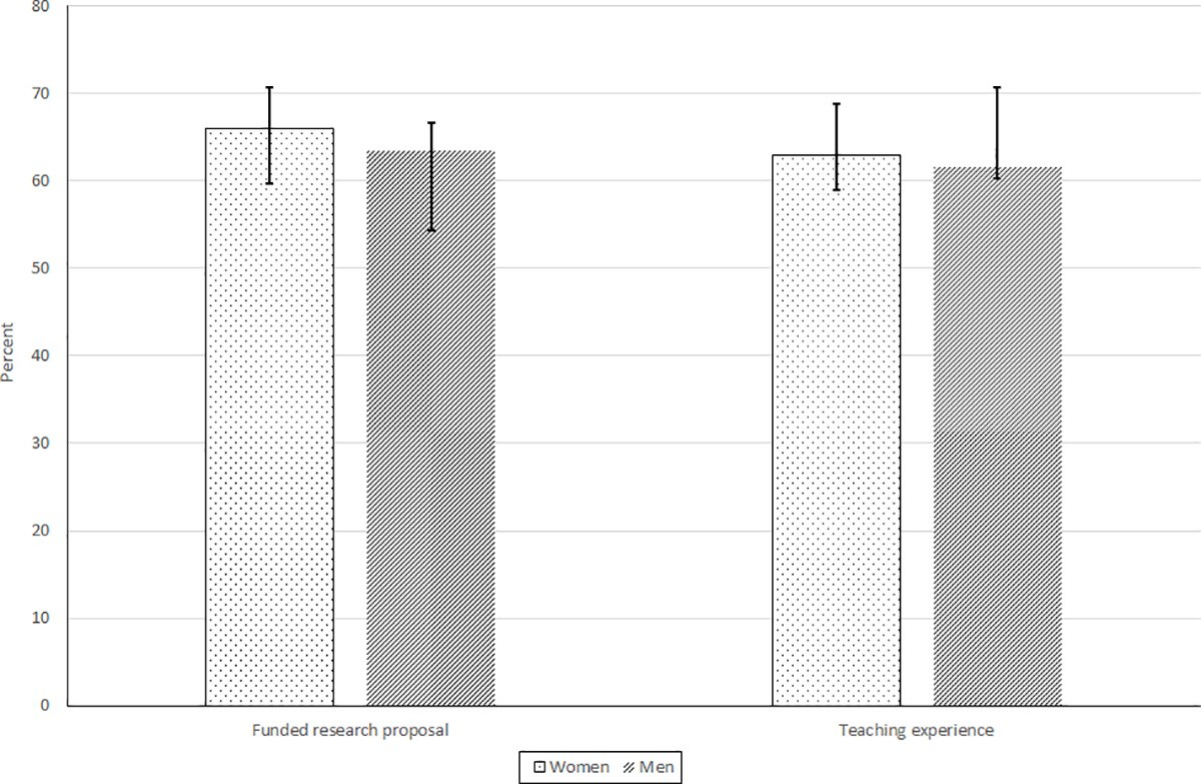
Percent having a funded research proposal and teaching experience during the PhD training: Sampled women and men at African universities (*n* = 227), 2005–2020. Source: RSIF gender survey, 2020.

**Fig 5:**
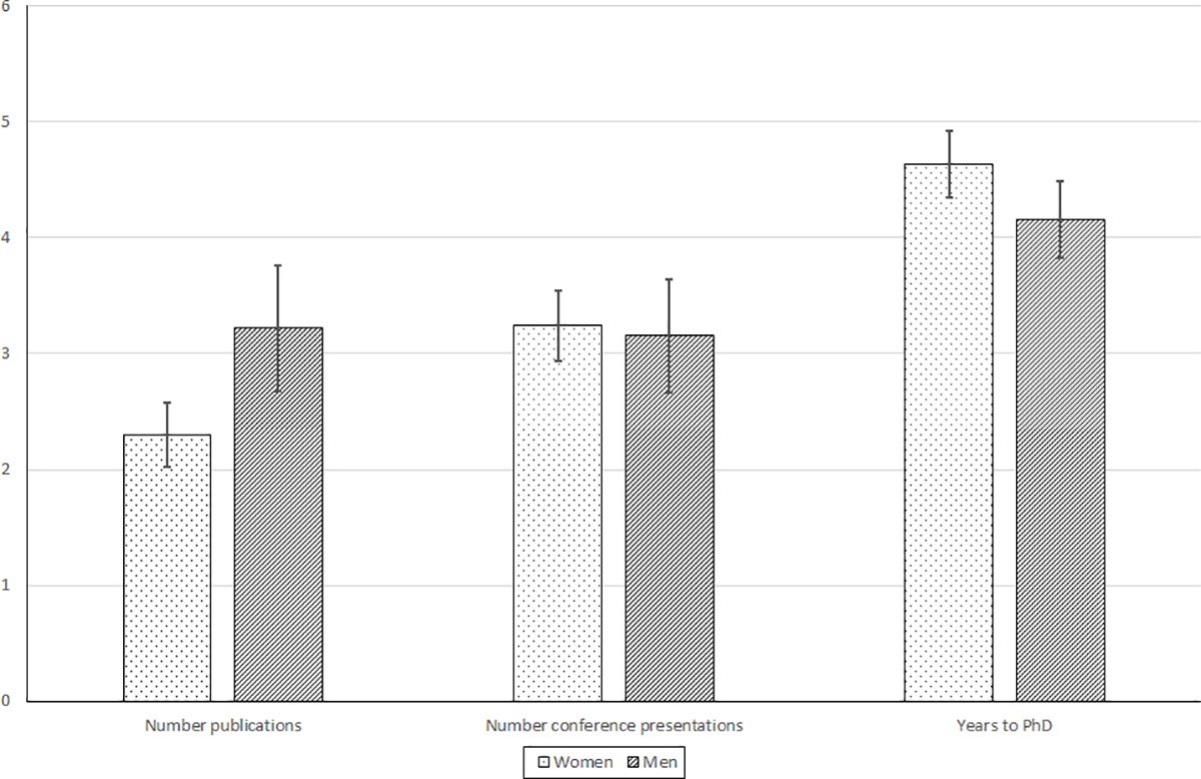
Number of accepted publications and conference presentations and years to PhD completion for sampled women and men at African universities (*n* = 227), 2005–2020. Source: RSIF gender survey, 2020.

Figs 4 and 5 present means/proportions and 95% confidence intervals for measures of PhD performance and completion. We find that, during their PhD training, sampled women had research proposals funded, gained teaching experience, and presented at conferences at similar rates to men (Figs 4 and 5). Qualitative interviews at four African institutions that were part of the larger RSIF gender study, support these findings with interviewed professors and department heads reporting that women make very good postgraduate students. However, compared to men, women on average had 0.91 (40%) fewer manuscripts accepted for publication during their PhD training.

**Fig 6:**
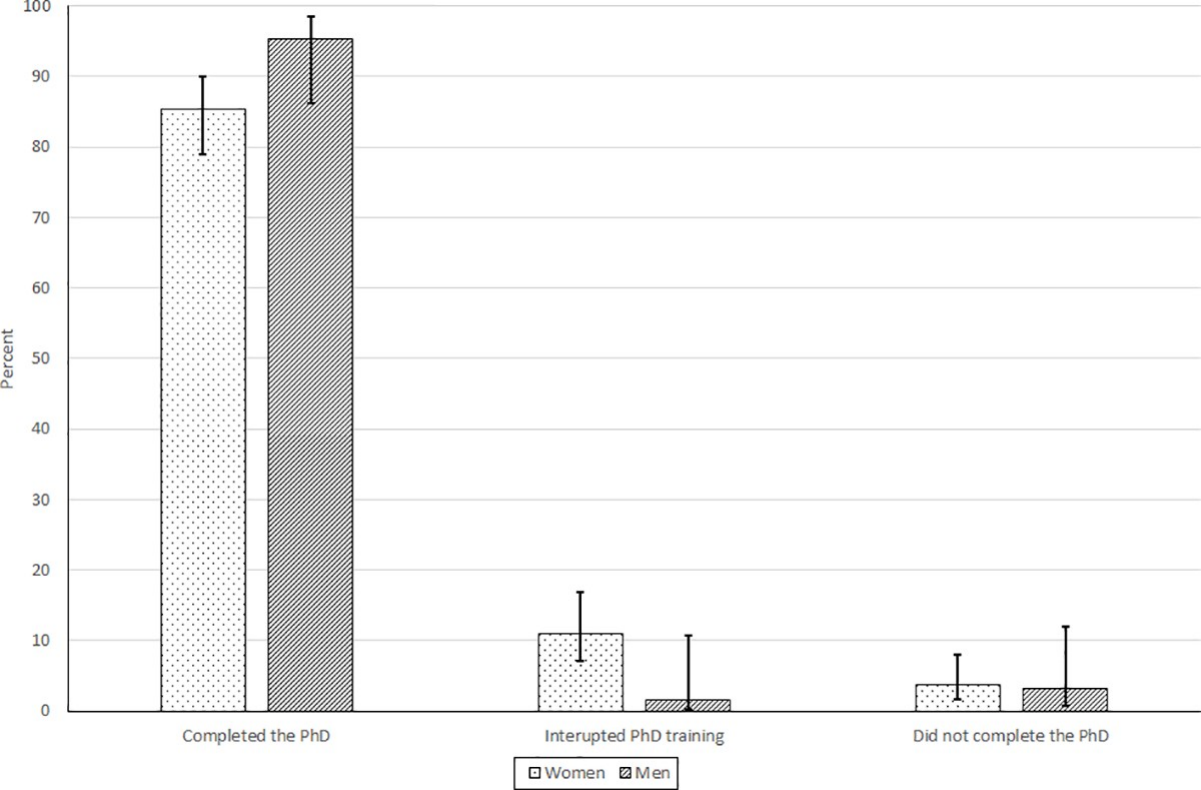
PhD completion rates at African universities for women and men (*n* = 227), 2005–2020. Source: RSIF gender survey, 2020.

In terms of PhD completion, survey findings indicate high PhD completion rates for women and men students alike (Fig 6). There are numerical differences, with men’s PhD completion rates exceeding those of women by 12%, and women students having a PhD interuption rate that was six times higher than that of men. Given our small sample size, we cannot rule out the possibility that the dataset does not have sufficient power to detect a true effect of gender on PhD completion. Sampled women on average took half a year longer to complete the PhD compared to men students, although this numerical difference is not statistically significant at the 0.05 level (Fig 5). Again, the small size of our sample calls into question the statistical power to detect a true effect.

### Regression results

Multicollinearity tests were run and an acceptable variance inflation factor (VIF) value of below 2.5 was attained for each explanatory variable revealing low correlation among variables being tested. Comparison of results for Poisson and negative binomial models revealed no significant differences, but due to the presence of over-dispersion we report results for the negative binomial model. To account for spatial dependence, the standard errors are clustered at the country level.

#### Pooled sample: Determinants of publication productivity

Table 2 presents negative binomial regression findings on the correlates of number of PhD publications accepted per year of PhD training. We ran four different models. Model 1 includes only the female binary variable as a predictor. Model 2 includes the full set of explanatory variables. We ran a series of regressions that added an interaction term between the female binary variable and each independent variable found statistically significant in Model 2. Only two such interaction terms are significant (*p* < 0.05) and these are included in Models 3 and 4.

**Table 2:** Negative binomial regression results for number of publications, pooled (women and men) sample (*n* = 224).

Explanatory variable	Model 1		Model 2		Model 3		Model 4	
	Coeff.	Std. Error	Coeff.	Std. Error	Coeff.	Std. Error	Coeff.	Std. Error
Constant			52.205**	31.055	51.575**	32.261	55.085**	31.785
Age at start of PhD (years)			-0.003	0.008	-0.004	0.008	-0.002	0.008
Female (0/1)	-0.430***	0.112	-0.306***	0.125	-0.209	0.162	0.149	0.208
Got married (0/1)			-0.353***	0.124	-0.063	0.178	-0.351***	0.124
Female*married					-0.444**	0.190		
Birth or adoption of child (0/1)			0.068	0.084	0.074	0.090	0.069	0.095
University/non-university funding (0/1)			0.112	0.113	0.124	0.121	0.118	0.117
Excellent research opportunity (0/1)			0.333***	0.111	0.317***	0.106	0.345***	0.104
Advisor provided guidance (0/1)			0.261**	0.112	0.258**	0.111	0.709***	0.136
Female*good guidance							-0.654***	0.204
Participated in a writing course (0/1)			0.206*	0.125	0.218*	0.118	0.204	0.132
Formal sciences (0/1)			0.102	0.163	0.106	0.169	0.133	0.165
Social sciences (0/1)			0.190	0.134	0.159	0.138	0.166	0.135
East Africa (0/1)			-0.252***	0.100	-0.264***	0.101	-0.286***	0.093
West or Central Africa (0/1)			-0.292**	0.124	-0.319**	0.135	-0.290***	0.110
Year left PhD training			-0.026*	0.015	-0.026	0.016	-0.028*	0.016

***, **, * indicate statistical significance at 1%, 5% and 10% levels, respectively.

For the base Model 1, the coefficient for the female variable is -0.430, which indicates that sampled women published 35% fewer papers per year of doctoral training compared to their male counterparts. As described by Cameron and Trivedi [32], negative binomial and poisson coefficients are converted to percentages by taking the exponential of the standard coefficients and multiplying by 100, in the case of positive coefficients. To obtain percentage figures in the case of negative coefficients, the exponential of the coefficient is subtracted from one then multiplied by 100.

Models 2, 3, and 4 indicate the following variables positively associate to publication output: an excellent research opportunity, an advisor that provided regular professional guidance, and participating in a writing course. Factors found to reduce publication productivity include getting married, pursuing the PhD at an institution in West or East Africa (reference category is southern Africa), and exiting the PhD program in a more recent year.

Comparing Models 1 and 2 reveals that including personal characteristics, family-related factors, funding, psycho-social variables, institutional factors, STEM discipline, region of the PhD institution, and PhD-exit year reduces the absolute value of the female effect and its statistical significance. Model 2 indicates women students had 26% fewer papers accepted for publication during their PhD training, compared to men students (*p* < 0.10).

Model 3 includes an interaction term between the female and got married binary variables. With the inclusion of this interaction term, there is no overall effect of being female or getting married. The latter finding along with statistical significance of the interaction term is indicative of a crossover interaction, meaning that the effect of getting married during the PhD training on publication productivity is opposite for women vs. men. Specifically, men who got married during their PhD studies published more, whereas women published less.

Model 4 adds to Model 2 an interaction term between the female binary variable and provision of regular professional guidance by the PhD supervisor. Interestingly, what the interaction term and the main effects indicate is that the positive influence of quality advising on publication output is higher for men vs. women PhD students.

#### Pooled sample: Determinants of years to PhD completion

Table 3 presents findings on variables that associate with the number of years taken to complete the PhD among the sub-sample of PhD completers (*n* = 190). Age at time of entry to the PhD program, being female, getting married, receiving university or non-university funding, and pursuing PhD training in West Africa are associated with longer time to PhD completion. Factors associated with faster PhD completion are having a personal goal for timely completion, if the university had an enforced time limit for PhD completion, and participation in an orientation program. Model 5 suggests that women took 12% longer to complete the PhD than men. Including the control variables in Model 6 slightly reduces the magnitude of this effect to 10%.

**Table 3:** Negative binomial regression results for number of years to PhD completion, pooled sample (*n* = 190).

Explanatory variable	Model 5		Model 6		Model 7		Model 8	
	Coeff.	Std. Error	Coeff.	Std. Error	Coeff.	Std. Error	Coeff.	Std. Error
Constant			0.151	9.643	-0.995	9.087	-0.311	9.472
Age at start of PhD (years)			0.010***	0.003	0.009***	0.003	0.010***	0.003
Female (0/1)	0.109***	0.042	0.094*	0.057	0.138*	0.074	0.034	0.048
Got married (0/1)			0.221***	0.059	0.347***	0.040	0.222***	0.063
Female*married					-0.173***	0.070		
Birth or adoption of a child (0/1)			0.052	0.084	0.055	0.083	-0.151***	0.059
Female*child							0.312**	0.130
University/non-university funding (0/1)			0.098*	0.053	0.100*	0.055	0.105*	0.055
Goal for timely PhD completion (0/1)			-0.135**	0.068	-0.137**	0.070	-0.144**	0.067
Advisor provided guidance (0/1)			-0.055	0.037	-0.056	0.038	-0.054	0.037
University time limit for PhD (0/1)			-0.087**	0.038	-0.090**	0.039	-0.085**	0.037
Participated in orientation (0/1)			-0.105***	0.040	-0.107**	0.045	-0.102***	0.037
Formal sciences (0/1)			-0.012	0.097	-0.009	0.095	-0.028	0.086
Social sciences (0/1)			-0.096	0.084	-0.103	0.084	-0.099	0.080
East Africa (0/1)			-0.006	0.065	-0.014	0.065	0.012	0.061
West or Central Africa (0/1)			0.101**	0.052	0.086	0.059	0.126**	0.052
Year left PhD training			0.001	0.005	0.001	0.004	0.001	0.005

***, **, * indicate statistical significance at 1%, 5% and 10% levels, respectively.

Models 7 and 8 (Table 3) include interaction terms between female and, alternately, getting married and having or adopting a child during the PhD training. The interaction term coefficient alongside the coefficient for the marriage variable indicates that getting married increased the time to PhD completion by 41% and 19% for men and women, respectively. The results for Model 8 suggest that having or adopting a young child during the PhD training increased completion time by 17% for women but reduced PhD duration for men by 14%.

#### Women’s sub-sample: Determinants of publication productivity and years to PhD completion

Results for the women’s sub-sample regressions are shown in Table 4. Only three variables are statistically significant in Model 9: getting married during the PhD training, having an excellent research opportunity, and participating in a writing course. The coefficient for the marriage variable indicates that getting married during the PhD decreased women’s publication output by 41%. There are some interesting results from Model 10 that could not be examined with the pooled sample regressions. Specifically, women took less time to complete the PhD if they had a female (vs. male) supervisor and if their PhD institution had policies that were supportive of women students and faculty. About 9% of women agreed or strongly agreed that sexual harassment by faculty was common in their PhD program (Table 1), and those women took 37% longer to complete the PhD than those who disagreed, strongly disagreed, or were neutral on the statement, as shown by findings for Model 10 (Table 4).

**Table 4:** Negative binomial regression results, women-only sample.

Explanatory variable	Model 9 (*n* = 162) Number publications		Model 10 (*n* = 133) Years PhD completion	
	Coeff.	Std. Error	Coeff.	Std. Error
Constant	40.168	33.402	-4.896	9.923
Age at start of PhD (years)	-0.004	0.012	0.005	0.004
Got married (0/1)	-0.529***	0.164	0.169**	0.074
Birth or adoption of a child (0/1)	-0.033	0.242	0.148	0.110
University/non-university funding (0/1)	0.039	0.130	0.157**	0.069
Excellent research opportunity (0/1)	0.294***	0.095		
Advisor provided guidance (0/1)	0.040	0.148	0.022	0.055
Female supervisor (0/1)	-0.026	0.122	-0.203***	0.079
Sexual harassment (0/1)	0.116	0.246	0.316*	0.167
Goal for timely PhD completion (0/1)			-0.186***	0.060
University time limit for PhD (0/1)			-0.086*	0.047
Participated in writing course (0/1)	0.262**	0.126		
Participated in orientation (0/1)			-0.079	0.063
University had gender policies (0/1)	0.074	0.200	-0.109**	0.046
Formal sciences (0/1)	0.227	0.317	-0.107	0.084
Social sciences (0/1)	0.218	0.143	-0.008	0.041
East Africa (0/1)	-0.070	0.162	0.057	0.103
West or Central Africa (0/1)	-0.071	0.150	0.156	0.108
Year left PhD training	-0.020	0.017	0.003	0.005

***, **, * indicate statistical significance at 1%, 5% and 10% levels, respectively.

## Discussion

### Synthesis of the study findings

Does a student’s gender influence PhD performance and time to PhD completion in STEM fields? Our study examined this question focusing on universities in sub-Saharan Africa (SSA) to begin addressing an important knowledge gap. The literature on women’s participation in STEM is voluminous for the case of North America and Europe. However, there is a paucity of research on gender-based differences in STEM for Africa. Influential commentaries and reviews by Tiedeu et al. [3] and Okeke et al. [2] stress the urgency of closing Africa’s gender gaps in STEM, and there are several insightful case studies [20,21]. However, lack of publicly available datasets from African institutions has translated to a paucity of quantitative or mixed-methods research on gender gaps in STEM in SSA.

Results of the present study indicate gender parity for some PhD performance measures: number of conference presentations and whether the student had a research proposal funded or gained teaching experience during the PhD training (Figs 4 and 5). However, student gender was found to matter for publication output, even when controlling for other implicating factors, with women PhD students reporting 26% fewer manuscripts accepted for publication in any given year, compared to their male counterparts. The observed gender disparity in publication output is relatively large. For instance, a study of six PhD student cohorts at the California Institute of Technology found that, during their doctoral studies, women published about 8.5% fewer papers than their male counterparts [27]. Mendoza-Denton et al. [26] found that 32% of women and 38% of men doctoral students in STEM fields at University of California Berkeley reported having submitted a paper for publication in the year prior to the survey.

The survey data reveals high PhD completion overall, with sizable numerical differences observed for the completion rate of men (95%) vs. women (85%), although not significant (*p* < 0.05). These PhD completion rates are relatively high, which might reflect that our sample over-represents individuals attending some of the most prestigious universities in Africa; we discuss this in more detail in the study limitations sub-section below. By comparison, the completion rate 10 years after U.S. students began PhD training was estimated by the Council of Graduate Schools at 54.7% for mathematics and physical sciences, 55.9% for social sciences, 62.9% for life sciences, and 63.6% for engineering. For our sample of women and men PhD alumni, PhD non-completion is essentially the same (3%), but a considerably higher percentage of women (11%) than men (2%) reported having to interrupt the PhD, which was usually for family reasons (e.g., getting married or giving birth). While these statistics are not significant at standard test levels, the small sample size calls into question whether we have a sufficiently large sample to detect effects. Previous research on gender differences in PhD completion is mixed. For instance, Okeke et al. [2] found no significant gender difference, whereas Jiang et al. [33] and Van de Schoot et al. [31] did observe significant differences. None of these studies concern SSA.

Our study reveals a statistically significant gender-based difference in the duration taken to complete the PhD training, with women taking 10% longer than men, controlling for key predictors. Previous studies in the United States and Europe reveal inconsistent findings on the association between gender and duration of the PhD trajectory. Maher et al. [29] found that women take longer than men to complete the PhD, although the balance of evidence did not reveal a gender effect [31,34,35].

Regression results suggest the factors affecting publication output and time to PhD completion are nearly identical for the sampled men and women PhD alumni. We found the following factors positively associate to publication output for women and men alike: having an excellent research opportunity, having a PhD advisor who provided regular professional guidance and was supportive of one’s goals, participating in a scientific writing course, and completing the PhD in southern Africa (vs. East and West Africa). Variables found to facilitate timely PhD completion for both women and men are starting the PhD training at a younger age, having a personal goal for timely completion, an enforced time limit for PhD completion at the institution, and attending an orientation program. Factors found to lengthen the time to PhD completion include having university or non-university funding and pursuing the PhD in West Africa (vs. southern Africa). These results generally agree with prior expectations and empirical evidence [29,36–40]. A counterintuitive finding is that having university or non-university funding lengthened the time to PhD completion among our sample. This result is not unprecedented, however. Horta et al. [28] reached a similar conclusion for their nationally representative sample of PhD holders in Portugal. Unfortunately, empirical studies for Africa that could serve as a benchmark do not exist, to our knowledge.

Why is it that, compared to sampled men, sampled women had lower publication output during the PhD training and took longer to complete their degree? Our findings implicate the advisor-student relationship, family factors, cultural factors, and systemic barriers. In terms of publication output, two gender-based differences were observed. First, having an excellent supervisor (who provided regular professional guidance and moral support), which was reported by about 20% of the surveyed women and men PhD alumni, associated to a 200% increase in men’s publication output but had a negligible (6%) impact on women’s publication output. This might seem puzzling at first glance, but there are several plausible hypotheses that merit future research. First, it may be the case that PhD supervisors push men students harder than women students to publish, if they subscribe to the gender stereotype that women are less capable than men in scientific fields. Second, it is conceivable that gender differences in thoroughness, cautiousness, and self-efficacy partly explain why the impact of good advising is less felt for women than men. Some research suggests that women STEM researchers are more thorough and cautious in publishing their work than their male counterparts [41], which may result in a smaller number of publications for women, although this may represent a tradeoff between quantity and quality. Self-efficacy is the belief in one’s capability to succeed in a domain [41]. Research has found that women tend to have lower self-efficacy for their abilities in STEM, compared to men [42,43]; lower self-efficacy may, in turn, make women more hesitant than men to submit their papers for evaluation [44]. Our study assessed self-efficacy of participants, but we could not include the variable in the regressions. This was because 100% of men had high self-efficacy (40% women), making this variable collinear with the female binary variable.

A second gender-based difference in the correlates of publication output is marriage. Getting married during the PhD training was found to reduce women’s publication productivity and increase that of men, which may reflect changes in domestic responsibilities upon marriage. Research consistently shows that marriage benefits men and disadvantages women, in terms of domestic labor responsibilities [45,46], which may downgrade women’s career ambitions, work productivity, and career progression [38]. Marriage can also present women with other forms of pressure that are not conducive to academic achievement. For example, 33% of the married women surveyed agreed or strongly agreed with the statement “During your PhD studies, you felt pressure to downplay your achievements and career prospects to avoid issues with your spouse (e.g., making him feel insecure)”.

Student gender was found to matter to PhD completion time in four main ways. First, having a child during the PhD training increased time to completion by 17% for women but reduced PhD duration for men by 14%. These findings agree with cross-country research showing that parenthood plays out differently for working men and women. Having children has been found to confer to men a “fatherhood bonus” (i.e., increased likelihood of being hired and higher pay) and to women a “motherhood penalty” (i.e., reduced likelihood of being hired and lower pay) [47]. These phenomena are partly explained by new mothers reducing their work hours, taking time off work, and/or seeking family-friendly work environments [48]; but these factors do not fully account for the female marriage penalty. Men, in contrast, have been observed to increase their work effort following the birth of their first child [48].

We found that having a woman supervisor reduced the time to PhD completion by 18% for sampled women alumni. Female supervisors can serve as important role models for women students, help counteract negative gender stereotypes that are pervasive in STEM, and provide students with a more favorable mentoring experience [2,49]. Consistent with the findings here, Canaan and Mougani [50] found that women undergraduate students in STEM at the American University of Beirut had higher GPAs and were more likely to graduate if paired with a woman supervisor. Interestingly, Pezzoni et al. [27] found that, on average, doctoral students in STEM published more when they had a female (vs. male) supervisor, but this premium applied to male students only.

Two key systemic barriers to women’s PhD completion were found in this study: limited gender policies and practices and sexual harassment by faculty at the survey institutions. Limited policies and practices to support women faculty and students at the universities was evident. Only 25% of surveyed women alumni had awareness of any policies and practices at their PhD institution to support women graduate students, such as maternity leave, on-site or subsidized childcare, or extension of academic deadlines. Importantly, women who said their university had such polices/practices in place finished their PhD in 18% less time than women who reported an absence of these policies/practices. About 9% of women agreed or strongly agreed that sexual harassment by faculty was common in their PhD program, and those women took 37% longer to complete the PhD than those who disagreed, strongly disagreed, or were neutral on the statement. Furthermore, less than half (45%) of surveyed women were aware of a sexual harassment policy at their PhD institution, and only 54% of these were familiar with the university’s reporting mechanisms for sexual harassment cases. This is just awareness, not if the policies actually are effective.

### Study limitations

There are some limitations of our study. First, the sample size is small relative to the area covered by the study. Second, our sample of PhD alumni were selected though non-probability sampling due to unavailability of a sample frame. To obtain our sample we contacted STEM faculty in our networks, organizations working to advance women in STEM, and PhD alumni that pursued their PhDs at RSIF African host institutions. Several of the programs we surveyed are World Bank African Centers of Excellence (ACE) programs that are relatively high performing, hence their selection as ACEs, and they typically have better research infrastructure and offer greater financial support to students, compared with other PhD programs in Africa. Our sample may therefore may not be generalizable to other African higher education institutions and PhD alumni. Third, the data are cross-sectional and rely on retrospective questions about the PhD experience, including the key outcome variables: number of publications accepted during the PhD training and number of years to PhD completion. Respondents that completed their PhD recently could more easily respond accurately to retrospective questions, but there is likely measurement error especially for respondents that exited the PhD program in 2000, the earliest exit year in the sample. Future research should identify ways to obtain a larger, randomly selected sample and collect information from PhD students when they enter and exit the program. The latter is planned for future cohorts of the RSIF PhD scholars.

### Policy implications

Two priority interventions emerge from the findings of this study: (1) family-friendly policies and facilities that are supportive of women’s roles as wives and mothers and (2) fostering broader linkages and networks for women in STEM, including ensuring mentoring and supervisory support that is tailored to their specific needs and circumstances.

There are best practices of family-friendly policies and facilities from the African continent that should be replicated. For example, the Consortium for Advanced Research Training in Africa (CARTA) covers the full costs of women doctoral fellows who are breastfeeding mothers to bring their child and a babysitter along for a month-long residential training seminar [20]. The program also allows fellows to stop the funding clock during their maternity leave, if they request it, with funding resuming upon their return to doctoral studies. Another example comes from Senegal, where a national program “Case des Tout-Petits” helps to ensure affordable and adequate childcare for children aged 0–6 years. These community-managed childcare facilities originally targeted rural localities and lower-income populations but have expanded to include universities, among others. For instance, University of Gaston-Berger in St. Louis, Senegal has such a childcare facility on campus, which is greatly appreciated by students and faculty that were interviewed as part of the larger RSIF gender study that included our survey. Female students also need to have awareness of the gender-responsive offerings at their universities, which could be partly achieved by including this information as standard content of an orientation program for incoming students. Finally, male champions have a critical role to play as agents of change in making marriage and motherhood compatible with being a productive scientist. Having a spouse who is emotionally supportive and takes an active role with household responsibilities could make a big difference in this regard, and there is need for male champions to help raise awareness and role model.

Facilitating an environment for women to expand their networks and engage with women role models/mentors can greatly increase women’s sense of belonging and their interest to continue their education and career transition in STEM fields, which are largely male dominated. There is a need to make women’s contributions more visible and normalize diversity in science, for example, by sponsoring women doctoral candidates to attend and present at conferences where women in science are key speakers, such as the Gender Summit Africa and the Global Forum for Women in Scientific Research. Mentorship and training programs such as The African Women in Agriculture Research and Development (AWARD), the Organization for Women in Science in the Developing World (OWSD), COACh-Cameroon, and Gender-responsive Researchers Equipped for Agricultural Transformation (GREAT) are promoting gender equality in education and research and increasing women’s opportunities to mentor or be mentored by another woman scientist. There is, however, a need for funding to enable widespread participation in these programs, which is especially important in disciplines, such as Physics, that have few female faculty.

Finally, it is important to promote a diversity of role models for women with STEM careers. Women who are single or who do not have children have needs that can be overlooked in the rush to support mothers and wives. Particuarly in institutional planning and orientations directed at students, it should be made clear that women scientists can live fulfilling lives that fall somewhat outside of social norms, such as the decision to remain unmarried or childless. The reality is that scientific careers are demanding and the choice to reduce one’s family obligations in order to focus on one’s primary interest should at least be part of the conversation about women’s lives.

### Implications for research

This study looks at an issue that has been drastically understudied and as such, it raises as many questions as it answers. What we can conclude is that, unsurprisingly, marriage and childbearing reduce the performance and slow the time to PhD completion for women, but not for men. Given that women often assume greater household responsibilities and childbearing has different consequences for them, the implication might be that women who want successful scientific careers might be better served by staying single and having no children, which should be a legitimate option. However, given the current low representation of women in STEM in Africa and elsewhere, many women are likely choosing marriage and parenthood over a STEM career.

A lingering question is what happens to women PhD candidates once they finish and move into the next stage of their careers? There is limited research capacity at African universities and research centers due to several factors, such as a shortage of research funding, an emphasis on teaching vs. research, and inadequate research facilities. Competition for academic and research positions is therefore considerable. Publications are the coin of the realm in STEM fields. One cannot have a career in science without regularly publishing. If marriage lowers women’s ability to publish during their PhD, there is no reason to think that would change upon graduation. In other words, if getting married reduces a woman’s tendency to publish, that challenge is ongoing and likely has long-term implications for a woman’s career in STEM. Further assessment using longitudinal data is needed to address the question of the extent to which women with families who complete PhDs are able to transition into and advance in a STEM career?.

The relatively high level of PhD completion among both men and women in SSA as opposed to other countries (e.g., the US) was not anticipated. There are at least three possible explanations. First, the universities and students sampled in our survey may not be representative, as highlighted earlier. PhD completion may be lower at non-surveyed African institutions. Second, PhD candidates in SSA may represent a far smaller percentage of the population in Africa than in the US. Those who survive the considerable hurdles of limited funding, smaller capacity in higher education and related issues, may simply represent the most qualified and impressive future scientists in their respective countries. Third, it is reasonable to ask what SSA universities may be doing that encourages retention in STEM doctoral programs. Is greater retention a product of better teaching, more comprehensive research support by professors, or better linkages between education and research training? In terms of the students, does earning a PhD in Africa have different implications for life course than is the case in western countries? For instance, do PhDs enjoy greater social support and admiration and are they more likely to be gainfully employed in the SSA context? Do any of these PhD impacts differ for male vs. female doctoral students? Clearly, findings of the present study suggest a large portfolio of important research questions for future social science research to inform policies for broadening participation in STEM in Africa.
